# Conformability in everolimus-eluting bioresorbable scaffolds compared with metal platform coronary stents in long lesions

**DOI:** 10.1007/s10554-017-1193-0

**Published:** 2017-07-06

**Authors:** Jiang Ming Fam, Yuki Ishibashi, Cordula Felix, Bu Chun Zhang, Roberto Diletti, Nicolas van Mieghem, Evelyn Regar, Ron van Domburg, Yoshinobu Onuma, Robert-Jan van Geuns

**Affiliations:** 1 Department of Cardiology, Thorax Centre, Room Ba-585, Erasmus University Medical Centre, ‘s-Gravendijkwal 230, 3015 GE Rotterdam, The Netherlands; 2 National Heart Centre, Singapore, Singapore; 3 The Affiliated Hospital of Xuzhou Medical College, Xuzhou, Jiangsu China

**Keywords:** Bioresorbable scaffolds, Conformability, Drug eluting stents, Long coronary lesions, Metallic platform stents, Percutaneous coronary Intervention

## Abstract

The aim of this study was to determine if there are significant differences in curvature of the treated vessel after the deployment of a polymeric BRS or MPS in long lesions. The impact of long polymeric bioresorbable scaffolds (BRS) compared with metallic platform stents (MPS) on vessel curvature is unknown. This retrospective study compares 32 patients who received a single everolimus-eluting BRS with 32 patients treated with a single MPS of 28 mm. Quantitative coronary angiography (QCA) was used to evaluate curvature of the treatment and peri-treatment region before and after percutaneous coronary intervention (PCI). Baseline demographic and angiographic characteristics were similar between the BRS and MPS groups. Pretreatment lesion length was 22.19 versus 20.38 mm in the BRS and MPS groups respectively (p = 0.803). After treatment, there was a decrease in median diastolic curvature in the MPS group (from 0.257 to 0.199 cm^−1^, p = 0.001). A similar trend was observed in the BRS group but did not reach statistical significance (median diastolic curvature from 0.305 to 0.283 cm^−1^, p = 0.056). Median Percentage relative change in diastolic curvature was lower in the BRS group compared with the MPS group (BRS vs. MPS: 7.48 vs. 29.4%, p = 0.013). By univariate analysis, use of MPS was an independent predictor of change in diastolic curvature (p = 0.022). In the deployment of long coronary scaffolds/stents (28 mm in length), BRS provides better conformability compared with MPS.

## Introduction

The everolimus-eluting bioresorbable scaffolds (BRS) represented a novel change in the treatment of coronary artery lesions. The BRS is composed of a poly-l-lactic acid (PLLA)—based platform. Besides the ability to have complete strut resorption at 36 months, there are several potential benefits of BRS including no trigger for thrombosis after resorption and restoration of vasoreactivity [1]. Typically, implantation of hard metallic implants straightens the coronary artery and thus modifies its curvature. A previous computational study demonstrated that after implantation of a metallic implant in a coronary artery, the curvature of the stent edges alters significantly which correlate to the changes in shear stress distribution and potentially with the neointimal proliferation pattern [2]. As implantation of coronary stents/scaffolds can alter blood rheology especially at the inflow and outflow edge of the stents, the vessel distortion post device implantation may contribute to early and late stent failure such as pertaining to stent fracture. Geometric changes in the arteries post implantation are largely determined by the conformability of the stent [3]. The conformability of the stent has been described as the flexibility of a stent in its expanded state with adaptation to the natural shape of the vessel. A higher conformability of the stent is associated with less potential for vessel distortion and trauma [4].

Previous studies using BRS in short lesions demonstrate better conformability and favorable clinical outcomes compared to MPS in the acute setting [5, 6]. In the study by Gomez Lara et al., the acute change in curvature and angulation as quantified by quantitative coronary angiographic analysis was decreased in BRS compared to MPS [6] and was shown to recover on follow up [7]. This effect may be more pronounced and more relevant in a long lesion in either the coronary or peripheral arterial system. However, the acute effects of its implantation on vessel geometry in long coronary lesions are yet to be investigated. The aim of this study was to determine if there are any significant differences in terms of curvature of the treated vessel after the deployment of a polymeric scaffold device in long lesions and compare this to a MPS.

## Methods

### Study design, population, and treatment device

This is a non-randomized, 2-arm, retrospective study performed with patients from the BVS Expand and BVS STEMI First registries that received a everolimus eluting BRS (ABSORB-BVS, Abbott Vascular, Santa Clara, CA, USA) compared with a subset of historical controls from the same institutional registries (X-SEARCH) who received a cobalt chromium- everolimus eluting stent (CoCr-EES; XIENCE^R^ stent, Abbott Vascular, Santa Clara, CA, US).

In brief, the common inclusion criteria for this study are patients who had received a single BRS or CoCr EES that are 28 mm in length in long coronary lesions. The patients in the BRS group are selected from the BVS Expand [8] and BVS STEMI [9] registries which are single centre prospective observational registries conducted at Thorax Centre, Erasmus Medical Centre that evaluates the long term safety and performance of the BRS-absorb coronary stent in routine clinical practice post market registration. Informed, written consent was obtained from the patients before they undergo any procedure. The lesions are also more complex with more bifurcations and calcified lesions. From the X-SEARCH registry, patients with similar angiographic characteristics were selected for this study [10].

**Fig. 1:**
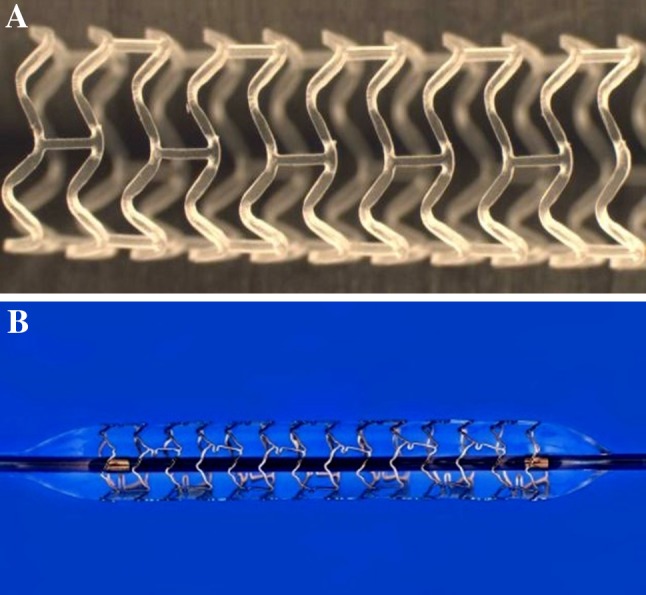
**a** Bioresorbable scaffold: The second generation ABSORB-BVS (Abbott Vascular, Santa Clara, CA, USA) has a strut thickness of 150 μm, consisting of in-phase zigzag hoops linked by bridges. The device is radiolucent but has two radioopaque platinum markers at each proximal and distal edge that facilitate ease of visualization on angiography. **b** Cobalt chromium everolimus- eluting stent (CoCr EES- XIENCE^R^, Abbott Vascular, Santa Clara, CA, US): The XIENCE^R^ are the metal platform stents and consist of a metallic platform made of cobalt chromium alloy. The struts are serpentine rings connected by links fabricated from a single piece. The XIENCE^R^ is covered by an everolimus coating

The BRS-Absorb vascular scaffold is a balloon-expandable device, consisting of a polymer backbone of PLLA coated with a thin layer of a 1:1 mixture of an amorphous matrix of PLLA polymer containing 100 μg/cm^2^ of the antiproliferative drug everolimus. The implant is radiolucent but has two platinum markers at each edge that allow visualization on angiography and other imaging modalities. Physically the scaffold has struts with an approximate thickness of 150 µm, which are arranged as in-phase zigzag hoops linked together by three longitudinal links (Fig. 1a).

The metallic platform of the everolimus-eluting XIENCE^R^ family stent (EES) is composed of a cobalt chromium (CoCr) alloy. The platform has a design similar to the Absorb platform and consists of serpentine rings connected by links fabricated from a single piece (Fig. 1b). The metallic platforms of the CoCr EES are constructed by a strut thickness of 81 µm each [11].

### Treatment procedure

Lesions treated with the BRS were implanted according to the procedural steps in line with the accepted recommendations at the time of the study. Predilation with either a semi-compliant or non-compliant balloon was highly encouraged. The BRS was implanted at a pressure not exceeding the rated burst pressure (16 atm). Post-dilation with either a semi-compliant or non-compliant balloon was performed at the discretion of the operator. Patients were prescribed with standard guideline recommended medical therapy including at least 12 months’ duration of dual antiplatelet therapy and antianginal therapy when appropriate.

### Quantitative coronary angiography (QCA) evaluation

Angiographic views with minimal foreshortening of the lesion and limited overlap with other vessels were used whenever possible for all phases of the treatment: preprocedural angiography, and after obtaining final result [12]. Comparison between pre and post treatment, were performed in matched angiographic views of 10° or less. The 2-dimensional (2D) angiograms were analyzed with the CASS 5.10 analysis system (Pie Medical BV, Maastricht, the Netherlands). In each patient, the treated region and the peri-treated regions (defined by 5 mm proximal and distal to the device edge) were analyzed. The computer defined minimal luminal diameter, reference diameter obtained by an interpolated method, and percentage diameter stenosis in the post procedure angiogram.

**Fig. 2:**
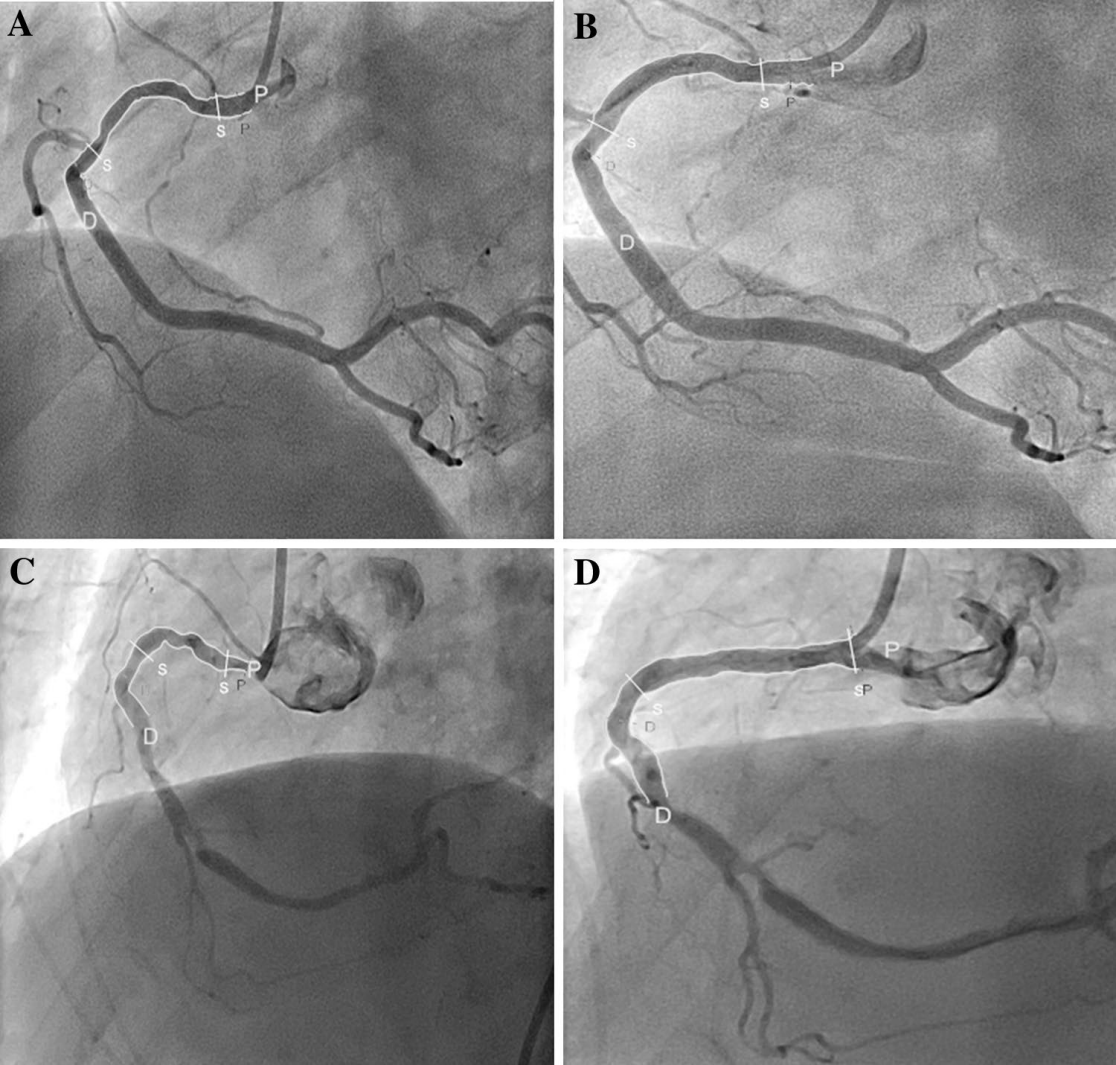
Curvature Analysis of the BRS and MPS: curvature analysis before and after deployment of a BRS (Fig. 2a, b) and a MPS (Fig. 2c, d). After implantation of a BRS, the curvature changed from 0.58 to 0.49 cm^−1^ whereas after the MPS was implanted, the curvature changed from 0.85 to 0.23 cm^−1^. *BRS* bioresorbable scaffold, *MPS* metallic platform stent

The definition of “Curvature” is the infinitesimal rate of change in the tangent vector at each point of the centerline. This measurement has a reciprocal relationship to the radius of the perfect circle defined by the curve at each point. The curvature of the vessel is calculated as 1/radius of the circle in cm^−1^, with a research program installed in the QCA Analysis software (CASS 5.10, Pie Medical Imaging) [13]. The segment of interest was defined as the stented/scaffolded length. To enable analysis of curvature in the same anatomical region, the scaffold position was superimposed on the preprocedural angiogram (Fig. 2). The software automatically detects the lumen contours of the selected segment and configures the centerline. Three points are then defined according to the centerline: one at the proximal, one at the distal, and one at the center of the defined segment. Next, a perfect circle is drawn through these points, calculating the radius of the circle and the curvature value. Prior to and after the procedure, the curvature of the segment of interest was repeatedly measured both during systole and diastole. Percentage relative change in curvature (Cv) was calculated as % (postCv–preCv)/preCv in the respective cardiac phases. Cyclic changes in vessel curvature were estimated as differences between systole and diastole at both pre-treatment and post-treatment.

### Statistical analysis

The Kolmogorov–Smirnov test was used to evaluate the normality assumptions of all continuous variables. Descriptive statistical analysis was performed with continuous variables expressed as median (interquartile range) and with categorical variables presented as counts (%). For comparison between groups, Mann–Whitney *U* test were used for the continuous variables. The Chi square test has been used to assess differences in categorical variables. Pre and post treatment comparisons within groups were assessed with Wilcoxon signed rank tests. Because the curvature, cyclic changes of curvature, and difference of curvature between pre- and post-treatment did not have a normal distribution, a log transformation was performed to achieve a normal distribution. A univariate analysis was performed between curvature and angulation changes with baseline demographic and angiographic variables. Variables that were found to be significant at the univariate level were tested with a multivariate linear regression model. (The thresholds for entry into and removal from the model were 0.1.) All statistical tests were carried out at the 5% level of significance. All analysis was performed by SPSS version 21 (SPSS, Inc., Chicago Illinois).

## Results

**Fig. 3:**
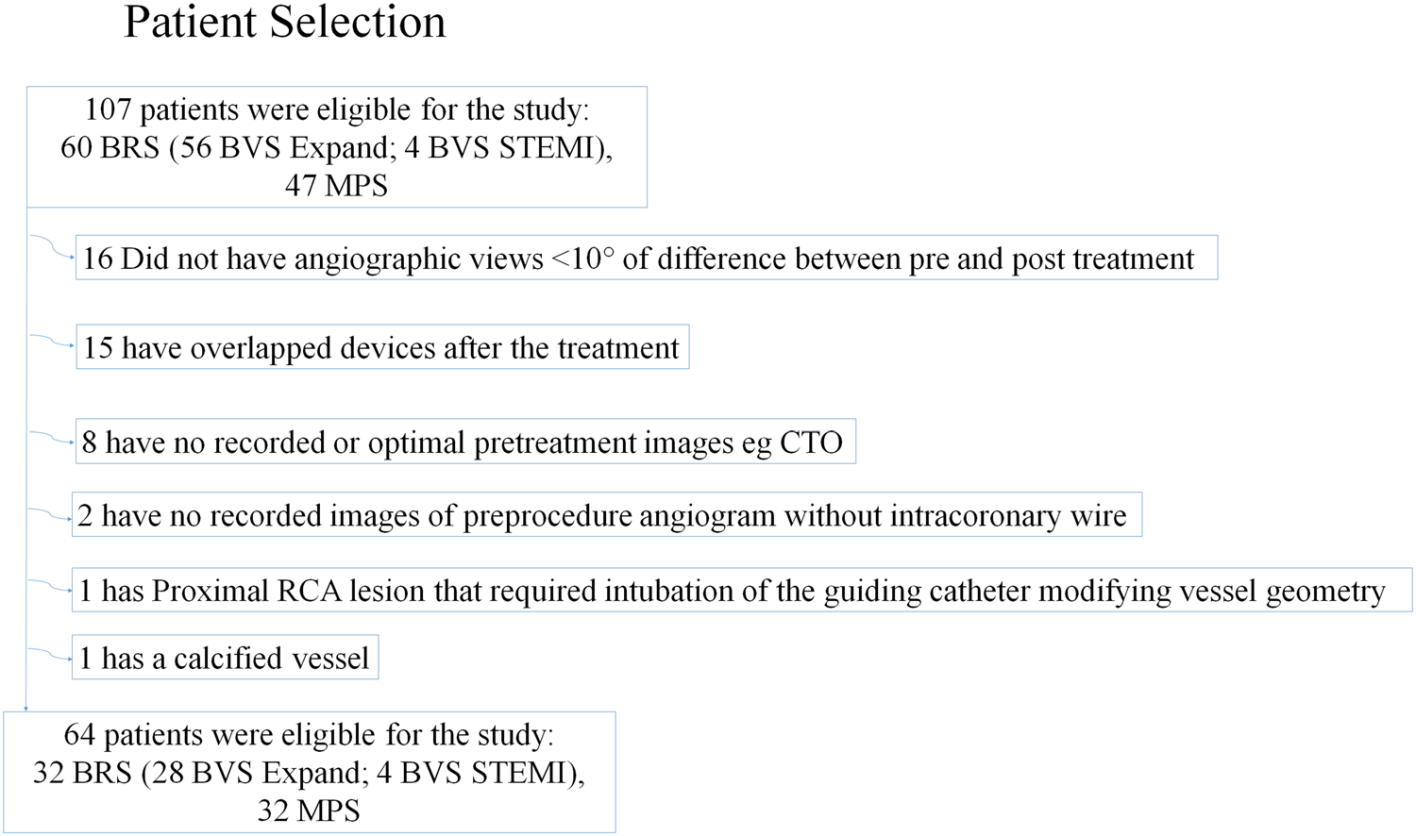
Flow chart of patient selection. *BRS* bioresorbable scaffold, *CTO* chronic total occlusion, *MPS* metallic platform stents, *STEMI* ST elevation myocardial infarct

The baseline clinical and angiographic characteristics are shown in Table 1. A total of 64 patients were involved in this study of which 32 were treated with the BRS and 32 with the MPS. A flow chart summarizing patient selection is shown in Fig. 3. There was no difference in median age (BRS vs. MPS: 59.6 vs. 64.9 years, p = 0.453), gender or clinical presentation between the 2 device groups. There were no significant differences in the cardiovascular risk factors.

**Table 1:** Baseline clinical and angiographic characteristics

	BRS (N = 32)	MPS (N = 32)	p value
Age (years)	59.6 (52.5, 67.8)	64.9 (57.7, 70.7)	0.453
Men	22 (68.8)	22 (68.8)	1.000
Hypertension	18 (56.2)	20 (62.5)	0.611
Hypercholesterolemia	15 (46.9)	17 (53.1)	0.617
Diabetes mellitus	5 (15.6)	8 (25.0)	0.351
Smoker (active)	12 (37.5)	7 (21.9)	0.391
Family history			
Previous CVA	2 (6.2)	2 (6.2)	1.000
Previous AMI	6 (18.8)	12 (37.5)	0.095
Previous PCI	5 (15.6)	9 (28.1)	0.226
Previous CABG	0	0	
Clinical presentation			
Stable or silent angina	10 (31.3)	18 (56.3)	0.074
Unstable angina	1 (3.1)	4 (12.5)	0.355
STEMI	4 (12.5)	0	0.155
NSTEMI	17 (53.1)	9 (28.1)	0.074
Other	0	1 (3.1)	1.000
Target vessel			0.857
LAD	15 (46.9)	13 (40.6)	
LCX	6 (18.8)	6 (18.8)	
RCA	11 (34.4)	13 (40.6)	
RVD (mm)	2.90 (2.49, 3.18)	2.91 (2.29, 3.26)	0.803
MLD (mm)	0.92 (0.77, 1.57)	1.20 (0.75, 1.55)	0.453
Diameter stenosis (%)	60.00 (47.25, 72.75)	56.00 (46.00, 76.75)	0.452
Bifurcation	12 (37.5)	7 (21.9)	0.274
AHA type			0.149
A	2 (6.3)	0	
B1	19 (59.4)	14 (43.8)	
B2	6 (18.8)	13 (40.6)	
C	5 (15.6)	5 (15.6)	
Calcification			
Mild	19 (59.4)	12 (37.5)	
Moderate/severe	13 (40.6)	20 (62.5)	
Pre-treatment region length (mm)	22.19 (17.67, 25.08)	20.38 (17.05, 25.75)	0.803
Procedural details			
Predilation performed	29	18	0.004
Predilation balloon diameter	2.50 (2.50, 2.50)	2.00 (2.00, 2.50)	0.03
Postdilation	18	13	0.317
Postdilation diameter	3.00 (2.94, 3.50)	3.50 (2.75, 4.00)	0.253

Values are presented as number (%) or median (interquartile range)

*AMI* acute myocardial infarct, *BRS* bioresorbable scaffold, *CABG* coronary artery bypass graft, *CVA* cerebrovascular accident, *LAD* left anterior descending artery, *LCX* left circumflex artery, *MLD* minimal luminal diameter, *MPS* metallic platform stent, *PCI* percutaneous coronary intervention, *RCA* right coronary artery, *RVD* reference vessel diameter, *STEMI* ST elevation myocardial infarct

The left anterior descending artery was the most commonly treated vessel in the study population. Lesion calcification and complexity were similar between the two groups (Table 1). Procedural data are as shown in Table 1. Lesions treated with BRS were predilated more frequently and at higher pressures compared to the lesions treated with metallic stents. Postdilation rates were similar. The pre treatment region length was 21.38 mm (17.67–25.58) in the overall group. There were no significant differences in reference vessel diameter, minimal lumen diameter and percentage diameter stenosis in both groups. Pretreatment curvature was similar between the BRS and MPS groups in both systole and diastole phases [systole: 0.290 (0.155–0.639) cm^−1^ vs. 0.283 (0.125–0.519) respectively, p = 0.803 and diastole: 0.305 (0.193–0.580) cm^−1^ vs. 0.257 (0.151–0.518) cm^−1^ respectively, p = 0.803].

### Geometric changes within and between groups

**Fig. 4:**
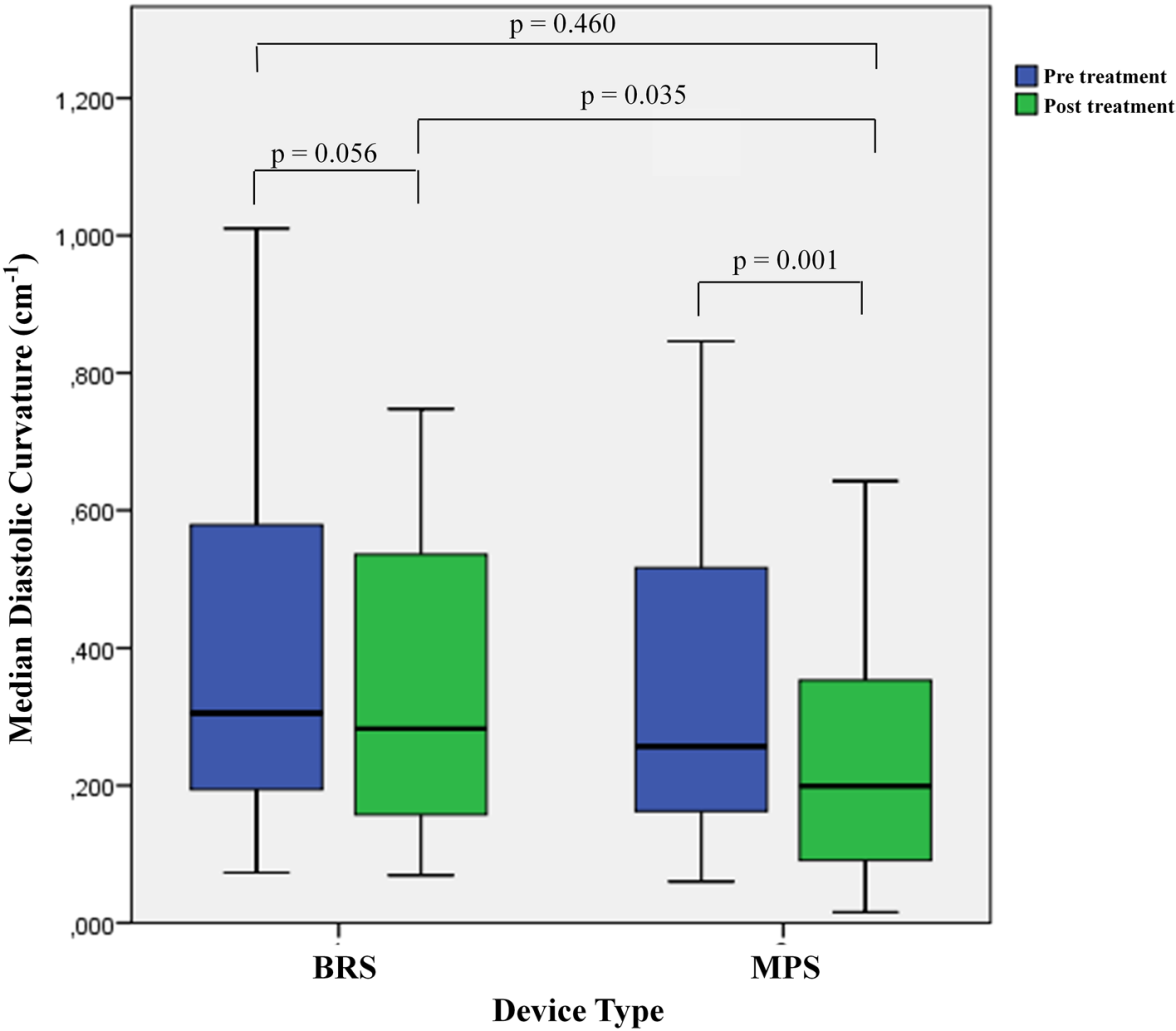
Change in curvature post treatment in BRS and MPS. This *boxplot* illustrates the difference in median diastolic curvature post treatment in the BRS compared to the MPS group

Table 2 shows the changes in curvature in both systole and diastole of the treated vessel in the BRS and MPS groups. After implantation of MPS, there was a significant decrease in median diastolic curvature (from 0.257 to 0.199 cm^−1^, p = 0.001) and median systolic curvature (0.283–0.194 cm^−1^, p < 0.001) representing a percentage reduction of 16.0 and 28.6% respectively. Following an absorb scaffold implantation, there was a trend towards a decrease in the median diastolic curvature (from 0.305 to 0.283 cm^−1^, p = 0.056) and median systolic curvature (from 0.290 to 0.282 cm^−1^, p = 0.061) which trends towards significance. As a result, the diastolic curvature was significantly higher in the BRS compared with the MPS group post treatment [BRS vs. MPS; 0.283 cm^−1^ (0.150–0.541) vs. 0.199 cm^−1^ (0.089–0.357), p = 0.035] (Fig. 4). Post treatment, Percentage relative reduction in curvature was also smaller in the BRS group compared with MPS group in both the diastole and systole phases [BRS vs. MPS; 7.48 vs. 29.4%, p = 0.013; 9.04 vs. 28.2%, p = 0.010 respectively]. Cyclic changes in curvature (i.e. between systole and diastole) were similar between the BRS and the MPS (p = 0.271).

**Table 2:** Changes in curvature of the study population

	BRS (N = 32)	MPS (N = 32)	p value
Pre-treatment curvature (cm^−1^)			
Systole	0.290 (0.155, 0.639)	0.283 (0.125, 0.519)	0.648
Diastole	0.305 (0.193, 0.580)	0.257 (0.151, 0.518)	0.460
Post-treatment curvature (cm^− 1^)			
Systole	0.282 (0.147, 0.549)	0.194 (0.097, 0.407)	0.077
Diastole	0.283 (0.150, 0.541)	0.199 (0.089, 0.357)	0.035
Percentage reduction in curvature post-pretreatment^a^			
Systole	2.76	28.6*	
Diastole	7.21	16.0*	
Absolute reduction in curvature (cm^−1^)			
Systole	0.024 (0.015, 0.087)	0.064 (0.010, 0.230)	0.034
Diastole	0.021 (0.025, 0.098)	0.090 (0.011, 0.192)	0.066
Percentage relative change in curvature (cm^−1^)			
Systole	−9.035 (−22.128, 7.911)	−28.17 (−46.22, −6.64)	0.010
Diastole	−7.484 (−23.193, 8.355)	−29.43 (−50.31, −3.55)	0.013
Pre-treatment cyclic change in curvature (cm^−1^)	−0.021 (−0.072, 0.061)	0.002 (−0.086, 0.096)	0.398
Post-treatment cyclic change in curvature (cm^−1^)	−0.026 (−0.054, 0.023)	−0.041 (−0.04, 0.125)	0.271

Values are presented as numbers or median (interquartile range)

^a^For BRS, the p values for comparison between pre and post curvature for systole and diastole are 0.061 and 0.056 respectively. For MPS, the p values for comparison between pre and post curvature for systole and diastole are <0.001(*) and 0.001(*) respectively

*BRS* bioresorbable scaffold, *MPS* metallic platform stent

### Predictive factors of modifying curvature

In univariate analysis, the use of MPS predicts a greater reduction in curvature with a coefficient of 23.33 (95% confidence interval 3.81–42.85, p = 0.02).

## Discussion

In summary, the major finding of this study showed that in the deployment of long coronary devices (28 mm in length), BRS showed a non-significant decrease in curvature in the post treated vessel compared with a significant reduction in curvature of the treated vessel with deployment of a MPS. Use of MPS was an independent predictor of vessel curvature change post deployment.

**Fig. 5:**
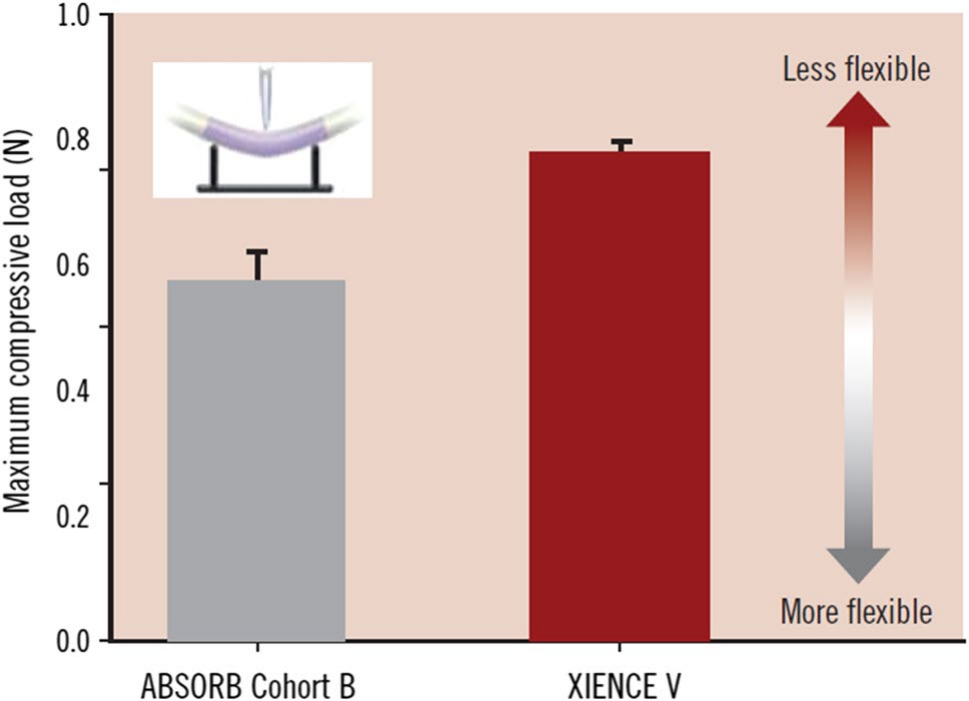
Maximum compressive force of ABSORB Cohort B scaffold and XIENCE V stent. This figure shows the maximum compressive force applied to deflect the ABSORB Cohort B and XIENCE V 3.0 × 18 mm devices by 1.1 mm using 3 point- bend test (n = 5). Statistical analysis yielded p = 0.004 using One- way ANOVA and Tukey- Kramer HSD. Tests were performed by and data are on file at Abbott Vascular. (Reprinted from EuroIntervention Supplement (2009) Vol.5 Supplement F; Oberhauser JP, Hossainy S, Rapoza RJ. Design principles and performance of bioresorbable polymeric vascular scaffolds. F15-22, Copyright, with permission from Europa Digital and Publishing)

Stent conformability is dependent on both the material and design of the stent and differs between the commercial devices that are available [14–16]. An open cell stent design would have higher conformability compared to a closed cell design. The difference in curvature post treatment between BRS and MPS could be attributed to the difference in underlying material composition of the devices in that a polymeric bioresorbable scaffold has better conformability to vessel geometry compared to metallic stents. In a study evaluating the bending stiffness of the BRS compared to the MPS in-vitro, the maximum compressive load of a BRS from ABSORB COHORT B trial was significantly lower compared to the XIENCE^R^ stent which signifies better conformability of the BRS (Fig. 5) [17]. This is despite the fact that the strut thickness of the ABSORB Cohort B stent is thicker than that of the XIENCE^R^ stent (strut thickness 152.4 vs. 81.3 µm). A previous study had shown that the use of relatively shorter (18 mm) BRS and MPS devices modify baseline vessel curvature but the change was more marked in the MPS compared with the BRS [6]. In this study, the median pretreatment lesion length was 16.3 and 16.8 mm in the BRS and MPS groups respectively which are comparatively shorter compared to our study population. To our knowledge, this is the first in vivo study that shown that BRS does not affect the curvature of the treated vessel significantly in the deployment of long scaffolds. This might be of useful significance as we treat longer lesions with overlap scaffold required.

Though OCT has been widely described in existing methodology [18–21] to evaluate scaffold performance, OCT by itself is not able to measure curvature of the vessel, whereas QCA is available pre and post in almost all patients. From fluid dynamics and the resulting shear stress we know curvatures do have an impact on plaque formation in the following years where it is important to minimize the distortion of the natural vessel course post stent or scaffold implantation. As vascular geometry is the most important determinant of local wall shear stress, any beneficial effect on the conformability of the blood vessel might have clinical implications. Studies have demonstrated that low wall shear stress promotes atherosclerosis and plaque progression in native arteries [22] and greater intimal hyperplasia after stent deployment [23]. Metallic stents deployed in curved porcine coronary arteries were noted to cause vessel straightening in the stented segment and increased curvature at the stent edges [2]. A study by Gyongyosi et al. had further showed that a longitudinal straightening of stents is an additional predictor of major adverse events [24]. There are possible physiological and clinical benefits arising from the improvement in conformability in the BRS. An increased conformability of the BRS platform may result in physiological wall shear stress at the stent edges due to less vessel distortion. This may translate to clinical benefits such as reduced risk of scaffold edge restenosis. However, the clinical benefits associated with better conformability still needs further evaluation. This has become more relevant in the setting of recent data that showed a potential lack of benefits up to 3 years [25] particular certain lesion subsets such as smaller vessels with the BRS compared with best in class DES, with the BRS showing either similar or increased risk of TLR and increased risks of scaffold thrombosis compared to DES [26, 27].

Stent flexibility (and conformability) is also one of the key determinants of stent fracture, a common cause of late stent failure. Hinge motion (i.e. rocking back and forth on a bend) was one of the factors that can increase the risk of stent strut fracture. Our results suggest that there is a subtle but certain cyclic change of curvature after device implantation in both groups. Although there is no difference between groups, one can speculate that this cyclic movement repeating greater than 86,400 times a day (based on average heart rate of 60 beats per minute) can cause mechanical failure at the metallic struts. In a study looking at predictors of stent fracture, stent fracture was identified in 2.9% of 1339 lesions treated with the XIENCE^R^ stent in only 6–9 months after placement [28]. In that study, the three major determinants of stent fracture in order of importance were hinge motion, ostial location and tortuosity. Since the BRS is programmed to get dismantled in the due course of the bioresorption, this might cause fewer problems with BRS than with MPS.

The impact of procedural factors such as predilation on conformability is still unknown. Although lesion pre and postdilation may potentially impact on outcome by its impact on lesion expansion (concentricity, eccentricity, final MLD/MLA, remaining DS% and AS%), changes in curvature is ultimately mostly influenced by the remaining implanted material characteristics and the design of the stent/scaffold (Number of longitudinal connectors). In clinical practice this is manifested by the straightening of the vessel during balloon inflations and increase in vessel curvatures directly after balloon deflation.

## Limitations

We acknowledge the following limitations. The study is non-randomized and population in each group is relatively small. 2D angiographic analysis may also not be the most optimal imaging modality to assess the geometry of coronary vessels. However the differences between the pre and post treatment angiographic views were less than 10°, indicating that the analysis were mainly performed in the same angiographic view. In addition, the precise impact of subsequent procedural steps (predilation, stent implantation, postdilation) on vascular curvature could not be entirely captured due to the inherent retrospective nature of our study and there was no specific protocol for operators to include the necessary angiographic or cinefluroscopic projections. Potentially this issue is best addressed in a future prospective study with dedicated research protocol ensuring the angiographic projections are obtained at the procedural steps of predilation, stent implantation and postdilation.

## Conclusion

In the deployment of long coronary scaffolds/stents (28 mm in length), bioresorbable scaffolds provides better conformability compared with MPS. The findings of this study and its clinical significance merits further evaluation.

## Abbreviations

AMI: Acute myocardial infarct
BRS: Bioresorbable scaffolds (BRS)
CABG: Coronary artery bypass graft
CoCr- EES: Cobalt chromium- everolimus eluting stent
Cv: Curvature
CVA: Cerebrovascular accident
DES: Drug eluting stent
LAD: Left anterior descending artery
LCX: Left circumflex artery
MLD: Minimal luminal diameter
MPS: Metallic platform stent
PCI: Percutaneous coronary intervention
PLLA: Poly-l-lactic acid
QCA: Quantitative coronary angiography
RCA: Right coronary artery
RVD: Reference vessel diameter
STEMI: ST Elevation myocardial infarct
